# A Review of the Current State of Off‐Label Therapies for Pediatric Inflammatory Bowel Disease

**DOI:** 10.1002/pdi3.70011

**Published:** 2025-06-17

**Authors:** Yassin EL‐Najjar, Mary‐Joe Touma, Shuai Tan, Xiaoqin Zhou, Qi Liu

**Affiliations:** ^1^ Department of Internal Medicine University of Texas Southwestern Medical Center Dallas Texas USA; ^2^ Department of Gastroenterology Children's Hospital of Chongqing Medical University National Clinical Research Center for Child Health and Disorders Ministry of Education Key Laboratory of Child Development and Disorders China International Science and Technology Cooperation Base of Child Development and Critical Disorders Chongqing Key Laboratory of Child Rare Diseases in Infection and Immunity Chongqing China

**Keywords:** biologic therapy, Crohn’s disease (CD), pediatric inflammatory bowel disease, small molecule, ulcerative colitis (UC)

## Abstract

Off‐label use of biologic therapies in patients with pediatric inflammatory bowel disease (IBD) has seen an increase in utilization. In this paper, we review the current state of off‐label therapies in the pediatric IBD population. Real‐world use of ustekinumab (UST), vedolizumab (VDZ), upadacitinib (UPA), tofacitinib, and ozanimod in the adult population could prove positive outcomes in the pediatric population. Established off‐label therapies inch closer to comparable safety, efficacy, and outcomes in pediatric IBD use. Outcomes and use of newer biologic therapies in patients with pediatric IBD have improved with increased rates of steroid‐free clinical remission (SFCR). Novel therapies, including Janus kinase (JAK) inhibitors and sphingosine‐1‐phosphate receptor (S1Pr) modulators, require further studies but could also prove effective.

## 1 Introduction

Inflammatory bowel diseases (IBDs) refer to a group of inflammatory intestinal diseases affecting pediatric and adult populations. The two main types of IBD include Crohn's disease (CD) and ulcerative colitis (UC). These disorders are characterized by periods of clinical and histological remission and relapse. Interactions between the host immune system, the environment, pathogens, and gut microbiome are responsible for the fluctuation of clinical status in a patient [1].

In the pediatric population, disease progression and status are even more critical due to the potential effect on milestones and overall child development. IBD in childhood and adolescence can present with a wider range of symptoms than adults. In some cases, particularly in early‐onset IBD (less than 6 years old), the disease can be more extensive at diagnosis and more aggressive in terms of abdominal pain, diarrhea, bleeding, weight loss, and extraintestinal manifestations [2].

Current treatments for IBD include immunomodulators, biologics, small molecules, and sphingosine‐1‐phosphate receptor (S1Pr) modulators. The approved use of these drugs differs by class and patient classification. Although some drugs have been approved by the FDA in adult and pediatric patient populations, some remain approved only for adult use or not yet approved for pediatric use. Although there has been a recent expansion in the list of therapies approved for treatment of IBD in adults, only two drugs, infliximab (IFX) and adalimumab (ADA), have been approved for the treatment of IBD in children at specific dosing schedules [3]. As a result, clinicians have increasingly turned to the off‐label use of other biologics and small molecules to manage pediatric IBD—either as monotherapy or, in select cases, as advanced combination therapies, such as the concurrent use of two biologics or a biologic with a small molecule agent. These off‐label treatment options are employed not only in some severe refractory cases but also when conventional treatments prove ineffective.

This review aims to provide a descriptive overview of the current landscape of off‐label biologics, small molecules, and S1Pr modulators in the treatment of pediatric IBD. The goal is to summarize emerging evidence drawn from published literature, without recommending specific treatment protocols or endorsing any therapeutic strategy.

## 2 Ustekinumab (UST)

Interleukin‐12 (IL‐12) and IL‐23 have been shown to play a significant role in intestinal homeostasis and inflammation. IL‐12 promotes the differentiation of naive T cells into T helper 1 (Th1) cells, which are responsible for the secretion of proinflammatory cytokines such as interferon‐gamma (IFN‐g). IL‐23 plays a crucial role in the expansion and maintenance of T helper 17 (Th17) cells, which in turn are responsible for the production of proinflammatory cytokines such as IL‐17A, IL‐17F, and IL‐22. IL‐12 and IL‐23 share structural similarity with both being heterodimeric cytokines that incorporate the p40 subunit. IL‐12 consists of p40 and p35 subunits, whereas IL‐23 consists of p40 and p19 subunits. The p40 subunit, common to both, is critical for the facilitation of receptor binding and downstream signaling [4]. UST, a human monoclonal immunoglobulin (Ig) G1 kappa monoclonal antibody, has emerged as an effective therapy for IBD as it targets the p40 subunit used by the IL‐12 and IL‐23 cytokines [5]. By inhibiting both IL‐12 and IL‐23, UST reduces Th1 and Th17 mediated inflammatory responses through dampened IFN‐γ production and decreased IL‐17/IL‐22‐driven inflammation.

In 2019, Dayan et al. highlighted the effective use of UST in a population of children and young adults with IBD. Fifty‐two patients with a median age of 16.8 years (81% CD, 8% UC, and 11% IBD‐unclassified [IBD‐U]), most of whom failed therapy with more than one anti‐tumor necrosis factor (anti‐TNF) (81%) or anti‐TNF and vedolizumab (VDZ, 37%), were analyzed for at least 12 months. Ten of the patients were also of biologic naiveness. The cohort was assessed retrospectively based on the Harvey Bradshaw Index or a partial Mayo score with the primary outcome being steroid‐free remission at 52 weeks. Their results showed that 39 patients (75%) were still on UST by week 52, with 50% of bioexposed and 90% of bionaive patients in steroid‐free clinical remission (SFCR). The group concluded that UST is effective and safe for induction and maintenance of clinical remission in pediatric patients with moderate to severe CD [6].

In 2021, the UniStar Phase 1 trial evaluated the pharmacokinetics, safety and efficacy of UST in children aged 2–18 years in the United States and 6–18 years worldwide. The study concluded that the drug profile was consistent with that observed in adults with CD; however, serum concentrations were found to be lower among patients with less than 40 kg body weight compared to those with body weight greater than 40 kg and the reference phase III adult population [7]. Phase 1 concluded with the clinical remission rate (defined as Pediatric Crohn's Disease Activity Index, PCDAI ≤ 10) of 22% and 29% at week 16 for the lower body weight and higher body weight groups, respectively. A long‐term extension (LTE) of the UniStar trial included 77% (34/44) patients who responded and entered LTE; 77% (26/34) and 24% (8/34) of patients, respectively, received UST at weeks 48 and 240. Clinical remission was achieved in 41.2% (14/34) of patients. Normalization of C‐reactive protein (CRP) was achieved in 29.2% (7/24) of patients and antibodies to UST were observed in 1 patient. At week 240, 1 or more adverse events (AEs) were reported in 91.2% (31/34) of patients and treatment discontinuation due to AEs was reported in 14.7% (5/34) of patients [8].

A systematic review conducted by Wang et al. pooled 11 studies, assessing data on 370 patients, with a primary outcome of clinical remission. Secondary outcomes included steroid‐free clinical remission, clinical response, endoscopic remission and improvement, safety, and change of UST dose. Among patients with CD, the primary outcome was achieved by 18.2%–42% of patients at 8–16 weeks and remission rates for maintenance therapy ranged from 30.3% to 66.67% at 52 weeks. For patients with UC, only one study was included and reported clinical remission rates of 60% at 52 weeks. Among secondary outcomes, it was worth noting that steroid‐free clinical remission rates among patients with CD ranged from 27.3% to 59.5% and 44% to 50% for patients with UC at 1 year. Six of the 11 studies that were included reported endoscopic remission rates of 0%–37.5% (*n* = 80 patients) and 63.6% (*n* = 11 patients) for patients with CD and UC, respectively. Eight of the 11 studies reported 0 serious AEs and the remaining 3 reported AE rates ranging from 4.5% to 20%. Overall, 5 out of 370 patients receiving UST discontinued the drug due to AEs with the most common being respiratory infection and headache [9].

In 2024, a multicenter retrospective study in China evaluated the clinical efficacy and safety of UST in children and adolescents with CD. 16 patients were included and 13 of them were followed for 1 year 11 of those 13 patients (85%) continued to receive UST after 1 year. The study showed remission rates of 41.7% at 24–32 weeks and 75% at 48–56 weeks, with Weighted Pediatric Crohn's Disease Activity Index (wPCDAI) scores lower than baseline at both intervals. Five children achieved complete endoscopic remission [10].

### 2.1 Dosing and Therapeutic Drug Monitoring

Dosing strategies in pediatric IBD off‐label use of UST are largely extrapolated from adult drug regimens. Induction typically involves a single intravenous (IV) dose of approximately 6 mg/kg, tailored to body weight. Maintenance therapy follows with subcutaneous injections, generally 90 mg every 8 weeks, though pediatric patients may require individualized adjustments. In cases of refractory disease or inadequate response, increasing the dosing frequency to every 4–6 weeks has demonstrated improved outcomes. The UniStar trial explored lower and higher IV induction doses in its study design, showing pharmacokinetic profiles of UST in pediatric patients similar to those of adult patients. Although no specific guideline delineates exact dosing, providers tend to adjust dosing for pediatric patients below 40 kg.

Therapeutic drug monitoring (TDM) is critical for optimizing UST therapy in pediatric populations. Measuring trough serum levels can guide dose adjustments, with target concentrations often exceeding 1–4 μg/mL, though specific thresholds may vary depending on disease severity and patient characteristics. Subtherapeutic levels are associated with reduced clinical efficacy, necessitating either dose escalation or interval shortening. Additionally, TDM helps identify and address immunogenicity, which can lead to the formation of antidrug antibodies and treatment failure [11].

## 3 VDZ

VDZ, a gut‐selective humanized monoclonal antibody used to treat IBD in adults by targeting the α4β7 integrin, is increasingly used off‐label to treat pediatric patients with IBD. The α4β7 integrin, expressed on the surface of T lymphocytes, binds to mucosal addressin cell adhesion molecule‐1 (MAdCAM‐1), which is selectively expressed on the endothelial cells of the gut‐associated vasculature. This interaction facilitates the adhesion and transmigration of T lymphocytes across the endothelium into the intestinal lamina propria, a process critical for lymphocyte homing to the gut. By blocking this pathway, VDZ prevents T lymphocyte infiltration into the intestinal mucosa, reducing localized inflammation while preserving systemic immune function, making it an effective and targeted therapy for pediatric IBD [12].

Retrospective cohort studies have been shown to be safe and effective in inducing and maintaining remission in children with IBD. VDZ is already recommended as a second‐line therapy in the most recent European Society for Paediatric Gastroenterology Hepatology and Nutrition and the European Crohn’s and Colitis Organization (ESPGHAN–ECCO) guidelines for pediatric UC (2018) [13] and CD (2020) [14].

A multicenter research group led by Dr. Jeffrey S Hyams reported on real world VDZ experience in 159 children and adolescents with IBD. VDZ was found to be safe and effective in treating pediatric patients with IBD, with approximately 43% achieving corticosteroid‐free clinical remission at 1 year in both CD and UC. No infusion related AEs were noted [15].

In an observational multicenter Spanish study, Garcia‐Romero et al. found that among 47 pediatric patients with IBD refractory to anti‐TNF drugs treated with VDZ, the clinical remission rate was 52.4% at week 14% and 52.8% at 30 weeks. Additionally, among patients in remission at week 14, 80% with CD and 84.5% with UC maintained remission at 52 weeks. AEs were uncommon and mild, with only a small percentage of patients experiencing headaches, alopecia, anemia, or dermatitis [16].

In 2022, a systematic review by Fang et al. evaluated the efficacy and safety of VDZ in children and adolescents with IBD. Ten studies were included with 455 patients. Notably, for CD, approximately, one‐third achieved clinical remission within 22 weeks, and about a half achieved remission at 1 year. Similarly, for UC or IBD‐U, comparable rates were observed. Mucosal healing occurred in patients with both CD and UC/IBD‐U. Serious AEs were reported in 6% of patients [17].

Shortly after, the HUBBLE study, an open‐label Phase 2 multicenter trial demonstrated the safety and efficacy of VDZ use in pediatric patients with moderate to severe UC or CD. Eighty‐nine patients were enrolled and randomized to different dosing according to weight (less than or more than 30 kg). In patients with UC, clinical remission rates across weight and dose groups at week 14 ranged from 20.0% to 38.5% on the Mayo score and from 30.0% to 61.5% on the Pediatric Ulcerative Colitis Activity Index (PUCAI). In patients with CD, clinical remission rates across weight and dose groups at week 14 ranged from 50.0% to 63.6% on the Crohn's Disease Activity Index (CDAI) and from 16.7% to 54.5% on the PCDAI [18].

In the study titled “Outcomes, dosing, and predictors of vedolizumab treatment in children with inflammatory bowel disease” (VEDOKIDS), in 2023, Atia et al. investigated the safety, effectiveness, and dosing of VDZ in children with IBD. This prospective multicenter cohort study enrolled 142 children, including 65 with CD, 68 with UC, and 9 with IBD‐U. Of note, children were managed according to local prescribing practices without standardized dosing. The primary outcome revealed that at 14 weeks, 42% of children with UC and 32% with CD achieved steroid‐free and exclusive enteral nutrition‐free remission. Atia and colleagues also found that VDZ serves as a safe and effective alternative for the 45 biologic‐naive patients. Interestingly, outcomes were even better than those observed in patients who had previously received biologics, further demonstrating the need to consider VDZ as an early intervention for patients with pediatric IBD [19].

### 3.1 Dosing and TDM

VDZ dosing in pediatric IBD typically involves weight‐based protocols adapted from adult regimens, with adjustments for age and body size. For patients weighing 30 kg or more, a standard adult dose of 300 mg is administered as an IV infusion at weeks 0, 2, and 6 during induction, followed by maintenance infusions every 8 weeks. For those weighing less than 30 kg, dosing typically involves IV infusions of 6 mg/kg or, in some cases, 10 mg/kg, based on physician's discretion and individual clinical considerations [19].

TDM has gained attention as a valuable tool in optimizing VDZ therapy. Recent data suggest that maintaining trough levels greater than 20 μg/mL at week 6 of induction and above 12 μg/mL during maintenance, as guided by TDM strategies used in adults, can enhance treatment durability and clinical outcomes. Moreover, TDM enables proactive adjustments to infusion intervals, reducing them to every 4–6 weeks when needed, which have been shown to improve therapeutic effectiveness [20]. These findings underscore the potential of TDM in refining VDZ therapy for pediatric patients while emphasizing the need for further research to establish pediatric‐specific guidelines for timing, frequency, and optimal therapeutic thresholds.

## 4 Janus Kinase (JAK) Inhibitors

JAK inhibitors are a class of medications that have emerged as a promising treatment option for IBD. These small‐molecule drugs work by targeting the JAK family of enzymes, which play a crucial role in the signaling pathways of cytokines and growth factors that mediate immune responses and inflammation. The mechanism of action of JAK inhibitors involves the inhibition of one or more of the JAK enzymes (JAK1, JAK2, JAK3, and TYK2) to disrupt the JAK‐signal transducer and activator of transcription (JAK‐STAT) pathway [21]. This interruption prevents the phosphorylation and activation of STAT proteins, which are essential for the transcription of pro‐inflammatory genes. Consequently, JAK inhibitors reduce the production of inflammatory cytokines and alleviate the immune response that contributes to the chronic inflammation characteristic of IBD [22]. JAK inhibitors have a fast onset of action and rapid modulation of proinflammatory pathways and can be useful for patients who need prompt disease management as well as long‐term maintenance. Although effective as monotherapy, JAK inhibitors can also be used in combination with other biologics as part of a dual‐therapy treatment regimen or as short‐term use in combination with other biologics [23].

In 2021, a cohort of 21 subjects, including 18 with UC or indeterminate IBD, were treated with tofacitinib. After a 12‐week induction period, 43% (9/21) demonstrated a clinical response and 33% (7/21) achieved steroid‐free remission. Following up at 52 weeks, 41% (7/17) maintained clinical response and steroid‐free remission and the patients continuing tofacitinib treatment for one year did not require steroid use. Tofacitinib was discontinued in nine subjects. Eight subjects due to refractory diseases, eventually underwent colectomy, and one discontinued due to an intra‐abdominal abscess. No instances of AEs were reported [24].

Two case reports regarding the use of JAK inhibitors in pediatric patients were reported, the first of which involved a 12‐year‐old female with severe refractory ileocolonic CD, unresponsive to multiple therapies including anti‐TNF, VDZ, UST, 6‐mercaptopurine, and ileocecectomy. The patient was hospitalized with severe symptoms and had missed school for over a month. PCDAI score was 45 (severe disease). Her condition minimally improved with steroid treatment, with a PCDAI score of 42.5 after 2 weeks. UST was discontinued, and treatment with upadacitinib (UPA) was started. Symptoms improved within 2 weeks, and she achieved clinical and biomarker remission (PCDAI score 0) within 4 weeks. By 8 weeks, she tapered off steroids and maintained remission status on a maintenance dose of 15 mg daily [25]. Another case involving a 13‐year‐old female with refractory UC, experienced persistent symptoms over a 6‐year period. Despite attempts with multiple biologic therapies, colonoscopies showed moderate inflammation (Mayo 2) and high calprotectin levels. UPA 45 mg daily was initiated, leading to symptom resolution within 1 week and normalization of calprotectin within 4 weeks. She maintained clinical remission without steroids for 9 months on a maintenance dose of 30 mg daily. The patient’s family opposed colectomy, opting to exhaust all medical therapy options [26].

A retrospective study from 14 centers affiliated with the Pediatric IBD Interest and Porto Groups of the European Society for Paediatric Gastroenterology Hepatology and Nutrition included children with IBD who underwent combinations of biologic agents or biologic and small molecule therapy for at least 3 months. The group evaluated dual biologic therapy effectiveness and safety in patients with pediatric IBD. Sixty‐two children (35 with CD and 27 with UC; median age 15.5 years) who had failed previous biologic treatments were included. Combinations included antitumor necrosis factor agents with VDZ (48%), UST (34%), or both (13%) and tofacitinib with a biologic (5%, *n* = 3). 2 patients were treated with combination of tofacitinib and IFX, and 1 patient was treated with tofacitinib and VDZ. The clinical response and remission rate of the patients treated with tofacitinib + biologic was 100% (clinical remission rates were 35% at 3 months, 50% at 6 months, and 63% at 12 months. CRP normalization and fecal calprotectin decrease to < 250 μg/g were achieved in 75% and 64% of patients at 12 months, respectively). AEs affected 47% of children, with 8 serious events leading to therapy discontinuation in 6 cases. 1 patient treated with tofacitinib, IFX, and corticosteroids developed a central catheter‐associated deep vein thrombosis [27]. The study concluded that dual biologic therapy shows promise for refractory pediatric IBD with appropriate risk considerations.

A single‐center retrospective case series evaluating the use of UPA in adolescents (12–17 years old) with IBD was conducted in 2023. Twenty patients, most of whom had failed multiple prior biologics, were treated with UPA for a median time of 51 weeks. UPA was used as monotherapy or in combination with other biologics. At 12 weeks, SFCR was achieved in 75% (15/20), with CRP normalization in 80% (16/20). Six‐month SFCR was maintained in 70% (14/20), and ultrasound monitoring showed response and remission rates of 77% at 12 weeks and 60% at 6 months. AEs were reported in 10% (2/20) and included cytomegalovirus (CMV) colitis and asymptomatic hyperlipidemia [28]. These findings suggest that UPA is effective in inducing and maintaining SFCR in adolescents with refractory IBD, supporting its potential use pending pediatric registration trials.

### 4.1 Dosing and Considerations

JAK inhibitors are characterized by predictable pharmacokinetics and standard oral dosing regimens. Typical induction and maintenance can also be tweaked depending on disease severity or the need for AE avoidance. Such a tailored approach could potentially optimize therapy, minimize toxicity, and improve overall disease control when standard dosing is insufficient. The use of JAK inhibitors for the treatment of pediatric IBD remains limited but continues to garner attention as retrospective studies in pediatric patients and wider use in adult patients grows. Clinicians must account for the increased vulnerability to infections in younger patients as well as dosing adjustments based on body weight and clinical response.

## 5 S1Pr Modulators

S1Pr modulators, such as ozanimod and etrasimod, have demonstrated safety and efficacy in adults with IBD. These agents primarily act by binding to S1Pr1, leading to internalization of the receptor on lymphocytes [29, 30]. By limiting lymphocyte egress from lymph nodes, they reduce migration into the systemic circulation potentially mitigating intestinal inflammation in IBD [30]. Although specific data on their apply in pediatric IBD are not yet available, some providers have initiated off‐label use of ozanimod for pediatric patients who have failed multiple biologics relying entirely on extrapolation from adult data. Further research, including retrospective cohorts, is needed to better understand the safety and efficacy of S1Pr modulators in this population.

### 5.1 Dosing and Considerations

Experience in younger patients remains limited, with caution being advised for potential cardiovascular effects (e.g., bradycardia and atrioventricular block) and increased risk of infection in a child's developing immune system. Weight‐based dosing strategies may be necessary to account for dynamic growth and maturation, and although TDM is not yet standard, some measures, such as obtaining lymphocyte counts and correlating with treatment response or side effects, could offer valuable insights. Close follow‐up is a necessity and should include regular cardiac evaluations and immunization review to ensure both safety and efficacy in the pediatric population.

## 6 Discussion

Small sample cohort studies will always lead the way to larger trials. Although there always remains a gap between adults and pediatric patients regarding drug approvals and data, having more available information using retrospective studies helps narrow the gap for providers looking to extend therapeutic options. The wider range of risks in conducting prospective clinical trials with a pediatric population includes a higher risk of harm, recruitment challenges, and ethical concerns related to the consenting process and shared decision‐making [31]. These risks, among others, influence the discrepancies between adults and pediatric treatment options.

The off‐label use of biologic therapies in pediatric IBD has been on the rise, reflecting a growing need to address the unique challenges faced by this population. Current evidence suggests that biologics, such as UST, VDZ, UPA, tofacitinib, and ozanimod, which have shown positive outcomes in adults, may offer similar benefits for pediatric patients. These therapies are gradually demonstrating comparable safety, efficacy, and outcomes in children with IBD, contributing to improved management of this disease. Notably, the increased use of these treatment options has been associated with higher rates of SFCR.

Despite these promising developments, further research is essential to fully understand the long‐term effects and optimal use of newer therapies in pediatric IBD. Novel treatments, including JAK inhibitors and S1Pr modulators, hold potential but require more extensive studies to confirm their efficacy and safety in children. As the landscape of pediatric IBD treatment evolves, ongoing clinical trials and real‐world data will be crucial in refining therapeutic strategies and ensuring that pediatric patients receive the most effective and safe treatments available.

## Author Contributions

Y.E.N and M.J.T conducted a review of the current literature on the topic and contributed equally to manuscript drafting and revision. S.T. was responsible for review and editing. X.Z. and Q.L were responsible for the conception, preparation, and revision of the manuscript. All the authors read and approved the final version of the manuscript before its submission.

## Ethics Statement

The authors have nothing to report.

## Conflicts of Interest

The authors declare no conflicts of interest.

## Data Availability

The authors have nothing to report.
